# 641 Utilization of Psychological Interventions by Burn Survivors with Depression and Anxiety Symptoms

**DOI:** 10.1093/jbcr/iraf019.270

**Published:** 2025-04-01

**Authors:** Kaitlyn Chacon, Kara McMullen, Edward Santos, Huan Deng, Dagmar Amtmann, Kimberly Roaten, Sarah Stoycos, Shelley Wiechman, Adrienne Taylor, Lewis Kazis, Jeffrey Schneider

**Affiliations:** Spaulding Rehabilitation Hospital, Harvard Medical School; University of Washington; Spaulding Rehabilitation Hospital; Spaulding Rehabilitation Hospital; University of Washington; University of Texas Southwestern; University of California Keck School of Medicine; Harborview Medical Center, University of Washington; Brigham and Women’s Hospital; Boston University; Spaulding Rehabilitation Hospital, Harvard Medical School

## Abstract

**Introduction:**

Depression and anxiety are common after burn injury. Psychological interventions are a key component in managing these mental health challenges. However, utilization of such interventions in the burn population is underexplored. Thus, this study aims to examine the association between psychological symptom severity and treatment utilization.

**Methods:**

The data of adult burn survivors from a multicenter longitudinal database from 2006 to 2024 were analyzed. The study sample was divided into two groups: minimal or no anxiety or depression symptoms (PROMIS Anxiety and Depression T < 60) and moderate to severe depression or anxiety symptoms (T ≥60) at 12 months. For between group comparisons, Wilcoxon-Mann-Whitney tests were applied for continuous variables, while Chi-square and Fisher’s exact tests were used for categorical variables. Logistic regression models assessed if psychological symptom severity was associated with receiving psychological interventions (psychological services at home or outpatient; psychological therapy or counseling; medication for pain, anxiety, or depression) at 12 months, controlling for demographic and clinical variables.

**Results:**

Out of 669 burn survivors assessed for symptoms of anxiety or depression at 12-months after injury, those in the moderate-to-severe symptom group (n=154), compared to the minimal symptom group (n=515), were younger (mean age 43 vs 49), female (72% vs 56%), unemployed at time of injury (30% vs 13%), single (70% vs 45%), have Medicaid insurance (28% vs 13%) and have a pre-injury psychiatric diagnosis (41% vs. 21%). The moderate-to-severe symptom group reported higher use of medication for pain, anxiety, or depression (89% vs. 63%), psychological therapy/counseling (34% vs. 12%) and home/outpatient psychological services (21% vs. 6%) at 12 months (p≤0.001 for all comparisons). Logistic regression analysis revealed that individuals with more severe symptoms were 171% more likely to receive psychological interventions at 12 months (p< 0.001). Additionally, those with larger burns had a 274% higher likelihood, and individuals treated at ‘Site 6’ were 321% more likely to receive these interventions (p< 0.001).

**Conclusions:**

Compared to those with no or mild symptoms, individuals with moderate or severe anxiety or depression symptoms were more likely to receive psychological interventions. Future work may examine the impact of treatment on those at high risk.

**Applicability of Research to Practice:**

This research emphasizes the importance of screening for anxiety and depression to ensure appropriate interventions are offered to individuals based on symptoms severity.

**Funding for the Study:**

NIDILRR #90DPBU0008, 90DPGE0004

